# Exceptional 106-month survival in multiply relapsed small cell lung cancer after sequential anlotinib and short-course sintilimab: a case report

**DOI:** 10.3389/fonc.2026.1902026

**Published:** 2026-09-09

**Authors:** Ruzhu Wang, Yishan Lu, Tianshu Yang, Yuting Zhang, Jingwen Chen, Ping Gao

**Affiliations:** 1 College of Health-Preservation and Wellness, Dalian Medical University, Dalian, China; 2 Department of Oncology, First Affiliated Hospital of Dalian Medical University, Dalian, China; 3 Department of Dermatology, First Affiliated Hospital of Dalian Medical University, Dalian, China

**Keywords:** anti-angiogenic therapy, case report, immune checkpoint inhibitor, long-term survival, small cell lung cancer

## Abstract

**Background:**

Small cell lung cancer (SCLC) is highly aggressive, and durable disease control after multiple treatment lines is uncommon. Anti-angiogenic therapy combined with PD-1 blockade may provide complementary antitumor effects through vascular and immune microenvironment remodeling.

**Case presentation:**

We report a 64-year-old never-smoking woman diagnosed with limited-stage SCLC in September 2017. She achieved a partial response after concurrent chemoradiotherapy but developed recurrence in November 2018 and subsequently progressed after irinotecan-based and pemetrexed-based chemotherapy. Anlotinib was initiated in August 2019 and achieved radiologic tumor regression. In May 2020, biopsy of a right submandibular lymph node confirmed extrathoracic metastatic SCLC. Sintilimab was added to ongoing anlotinib in June 2020, and a partial response was achieved after three cycles. Grade 2 immune-related hypothyroidism developed during combination therapy, and sintilimab was discontinued. Anlotinib monotherapy was subsequently continued as maintenance treatment. At the latest follow-up in July 2026, the patient remained alive with stable disease, with an overall survival of 106 months from initial diagnosis.

**Conclusions:**

This case highlights exceptionally durable disease control in multiply relapsed SCLC following sequential anlotinib and short-course sintilimab. The sustained activity of anlotinib, reactivation of antitumor immunity by PD-1 blockade, and potential vascular–immune interaction may have jointly contributed to the prolonged outcome. However, because anlotinib had demonstrated activity before immunotherapy and was continued after sintilimab discontinuation, the relative contribution of each treatment cannot be determined. Further prospective studies with biomarker analyses are warranted.

## 1 Introduction

Small cell lung cancer (SCLC) is a highly aggressive neuroendocrine tumor, accounting for approximately 15–20% of all lung cancers (1). It is characterized by rapid proliferation, a propensity for early distant metastasis, and high sensitivity to initial chemoradiotherapy; however, it also demonstrates a strong tendency for relapse (2). Recently, multiple phase III clinical trials have indicated that, in the first-line treatment of extensive-stage small cell lung cancer, the combination of immune checkpoint inhibitors (ICIs) with platinum-based chemotherapy can extend overall survival compared to chemotherapy alone (3, 4). However, despite these advances, most patients with extensive-stage SCLC eventually experience disease progression. This suggests that reliance solely on immunotherapy in conjunction with chemotherapy is insufficient for achieving durable disease control, and durable disease control after multiple lines of treatment remains uncommon.

Anti-angiogenic therapy may enhance the activity of programmed cell death protein 1/programmed death-ligand 1 (PD-1/PD-L1) blockade through vascular normalization, improved immune cell trafficking, and remodeling of the tumor microenvironment (5). The multi-target tyrosine kinase inhibitor anlotinib simultaneously targets multiple signaling pathways, including vascular endothelial growth factor receptor (VEGFR), fibroblast growth factor receptor (FGFR), and platelet-derived growth factor receptor (PDGFR). Prior studies have shown that anlotinib can prolong progression-free survival while maintaining a manageable safety profile in patients with SCLC who have received third-line or later treatment. Moreover, preclinical evidence indicates that it may improve immune cell infiltration when combined with immunotherapy. However, long-term real-world evidence remains limited, especially after the failure of multiple prior regimens (6, 7).

Here, we report a patient with multiply relapsed SCLC who achieved a partial response (PR) and subsequent durable stable disease (SD) after sintilimab plus anlotinib, followed by anlotinib maintenance. This case may help refine clinical thinking regarding patient selection, timing of combination treatment, and the potential role of maintenance anti-angiogenic therapy in later-line SCLC.

## 2 Case description

### 2.1 Patient information

The patient was a 64-year-old never-smoking woman with no remarkable past medical history and no known family history of malignancy. In September 2017, a right hilar mass was incidentally detected during a routine health examination, and she was referred to our center for further evaluation.

### 2.2 Clinical findings

**Figure 1:**
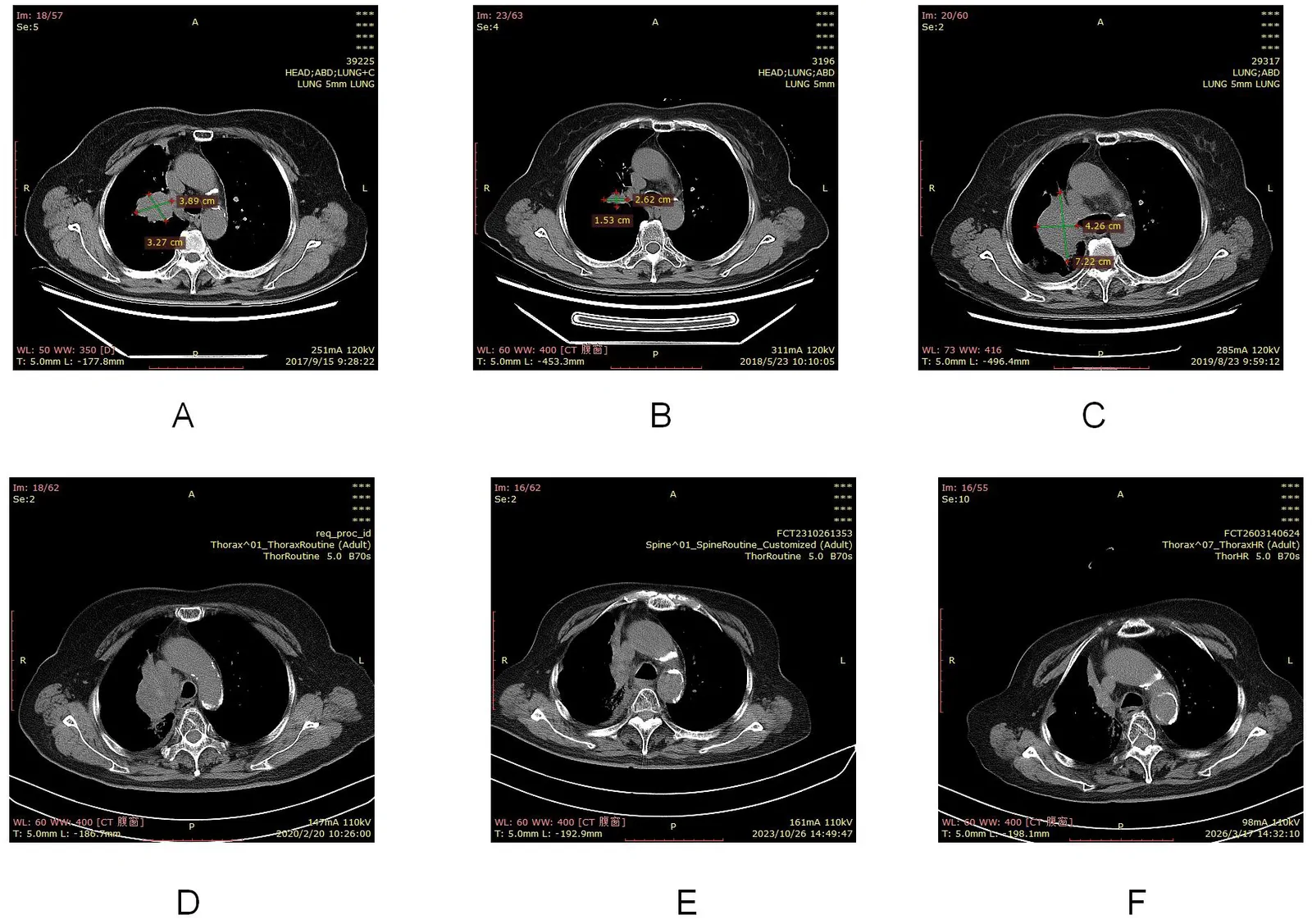
Serial chest computed tomography images during the disease course. **(A)** Baseline chest computed tomography in September 2017 showing a right hilar mass with mediastinal lymph node enlargement. **(B)** Post-treatment imaging after concurrent chemoradiotherapy showing PR. **(C)** Imaging at disease recurrence after subsequent chemotherapy. **(D)** Tumor regression after anlotinib treatment, before biopsy-confirmed extrathoracic progression. **(E)** Follow-up chest CT in October 2023 showing stable disease during anlotinib maintenance after discontinuation of sintilimab. **(F)** Latest available follow-up imaging in March 2026 showing ongoing SD.

Physical examination was unremarkable at initial presentation. Chest computed tomography (CT) revealed an irregular soft tissue mass in the right hilar region, measuring approximately 3.5×2.8 cm, along with mild enlargement of the mediastinal lymph nodes (**Figure 1A**). Brain magnetic resonance imaging (MRI) and abdominal CT scans showed no evidence of distant metastasis.

In May 2020, the patient noted a painless swelling in the right submandibular region. Physical examination revealed a palpable, mobile, non-tender right submandibular lymph node measuring approximately 2 cm in diameter.

### 2.3 Diagnostic assessment

**Figure 2:**
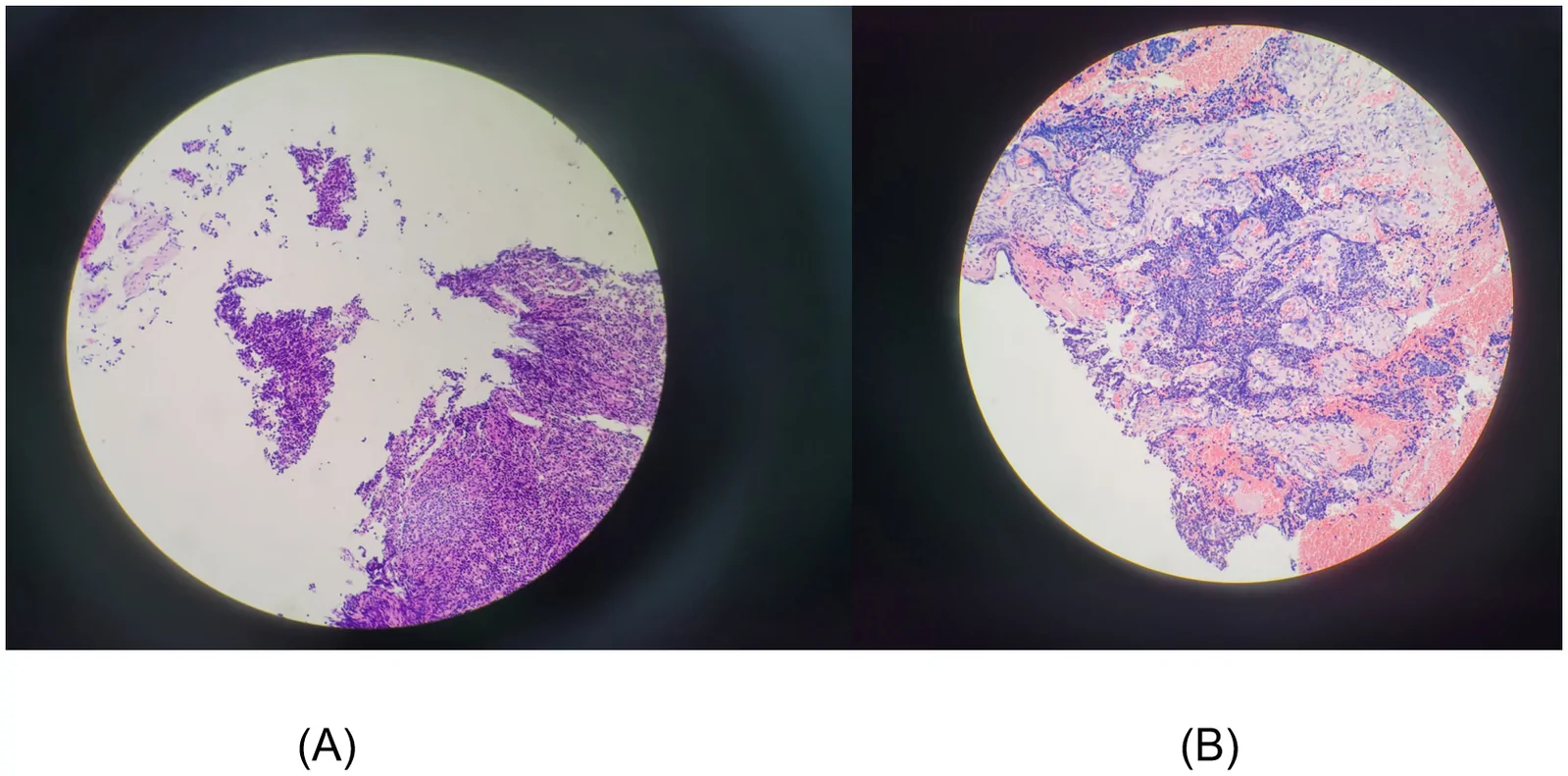
Histopathological findings of the primary tumor and metastatic lymph node. **(A)** Hematoxylin and eosin (H&E)-stained bronchoscopic biopsy specimen from the right hilar lesion obtained in September 2017, showing morphology consistent with small cell lung cancer (original magnification ×300). **(B)** H&E-stained ultrasound-guided needle biopsy specimen from the right submandibular lymph node obtained in May 2020, confirming metastatic small cell lung cancer (original magnification ×300).

A bronchoscopic biopsy confirmed the diagnosis of SCLC (**Figure 2A**). Immunohistochemical analysis demonstrated positivity for cluster of differentiation 56 (CD56), partial positivity for cytokeratin (CK), and positivity for chromogranin A (CgA), synaptophysin (Syn), and thyroid transcription factor-1 (TTF-1), thereby indicating a neuroendocrine origin. Based on the comprehensive imaging and pathological findings, a diagnosis of limited-stage small cell lung cancer (LS-SCLC) was established.

In May 2020, ultrasound-guided core needle biopsy of the right submandibular lymph node confirmed metastatic SCLC, indicating progression to ES-SCLC (**Figure 2B**).

### 2.4 Therapeutic intervention

**Figure 3:**
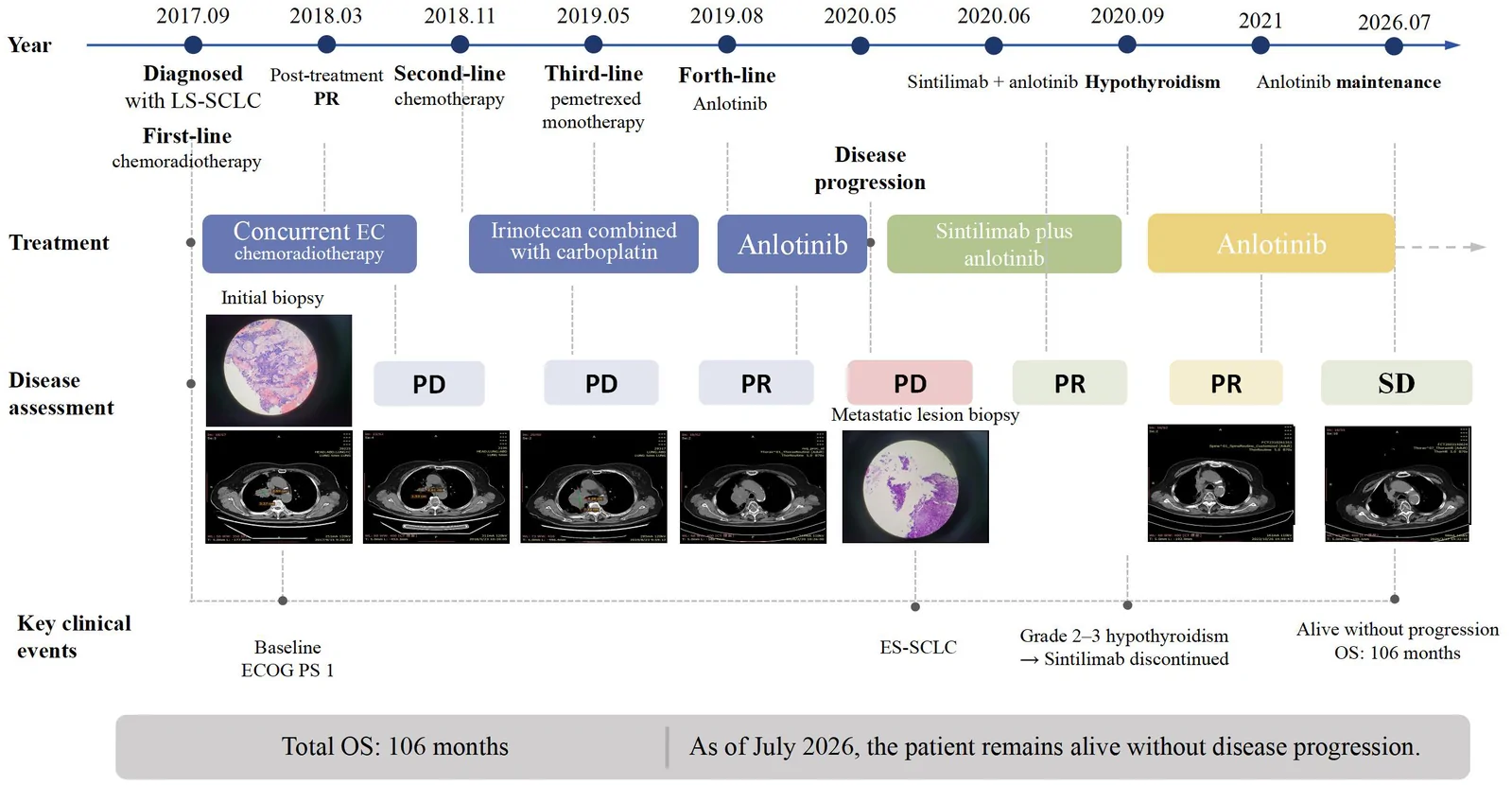
Timeline of diagnosis, treatment, response, and adverse event management. The patient was diagnosed with limited-stage small cell lung cancer in September 2017 and received etoposide plus carboplatin with concurrent thoracic radiotherapy beginning in October 2017, achieving a PR. Disease recurrence was documented in November 2018, followed by irinotecan-based chemotherapy and pemetrexed without durable benefit. Anlotinib was initiated in August 2019 and was associated with radiologic tumor regression. Biopsy-proven right submandibular lymph node metastasis in May 2020 indicated progression to extensive-stage disease. Sintilimab plus anlotinib was initiated in June 2020. After three cycles, radiological assessment demonstrated a PR, after which sintilimab was discontinued and anlotinib monotherapy was continued as maintenance treatment. Grade 2 hypothyroidism developed during treatment and was controlled with levothyroxine. At the latest clinical follow-up in July 2026, the patient remained alive without clinically documented disease progression.

Beginning in October 2017, the patient received treatment with etoposide in combination with carboplatin and concurrent thoracic radiotherapy, which delivered a total dose of 60 Gy in 30 fractions. Post-treatment evaluations demonstrated a significant reduction in thoracic lesions, with efficacy assessment indicating a PR (**Figure 1B**). During this treatment period, no grade ≥3 treatment-related adverse events were reported. The treatment course is summarized in **Figure 3**.

During follow-up in November 2018, the lesions in the right hilum and mediastinum showed enlargement, indicating disease recurrence. The patient subsequently underwent irinotecan-based chemotherapy followed by pemetrexed therapy; however, neither regimen produced durable disease control (**Figure 1C**).

Given the limited benefit from multiple chemotherapy regimens and the patient’s preserved ECOG performance status, oral anlotinib was initiated in August 2019. Treatment was associated with significant radiologic regression of the thoracic lesions (**Figure 1D**), suggesting that the tumor retained sensitivity to anti-angiogenic therapy despite progression after several previous lines of treatment.

After biopsy confirmation of extrathoracic metastasis in May 2020, and considering the previously demonstrated clinical benefit of anlotinib, the relatively limited metastatic burden, and the patient’s preserved performance status, treatment with sintilimab (200 mg on day 1, every 21 days) in combination with anlotinib (12 mg once daily on days 1–14, every 21 days) was initiated in June 2020.

After three cycles of sintilimab plus anlotinib, radiologic assessment showed further shrinkage of the thoracic lesions, with a best overall response of PR according to RECIST version 1.1. During combination treatment, the patient developed grade 2 immune-related hypothyroidism, which was well controlled with levothyroxine and did not mandate immediate treatment interruption; nevertheless, the potential risk of additional cumulative immune-related toxicity was considered during shared decision-making regarding treatment duration. After completion of three cycles, sintilimab was discontinued, and anlotinib monotherapy was continued as maintenance treatment. This decision was based on the observed tumor response, the lack of prospective evidence defining the optimal duration of PD-1 blockade in relapsed SCLC, concern regarding potential cumulative immune-related toxicity, the patient’s previous clinical benefit from anlotinib, and her preference for long-term oral maintenance therapy rather than continued intravenous immunotherapy. The patient subsequently underwent close clinical and radiological surveillance.

### 2.5 Follow-up and outcomes

During long-term follow-up on anlotinib maintenance, imaging showed no new lesions, and both the primary tumor and metastasis-related lesions remained consistently stable (SD) (**Figure 1E**). Throughout her treatment course, the patient did not experience severe immune-related adverse events such as immune pneumonitis or immune hepatitis. The grade 2 hypothyroidism remained well controlled with levothyroxine replacement and did not interfere with subsequent anlotinib maintenance treatment or the patient’s daily activities. Throughout the disease course, including periods of disease progression and multiple lines of systemic therapy, the patient’s ECOG performance status remained between 0 and 1, allowing continuation of subsequent treatments without significant deterioration in functional capacity.

The latest imaging assessment in March 2026 demonstrated ongoing SD (**Figure 1F**). At the most recent clinical follow-up in July 2026, the patient remained alive without clinically documented disease progression, corresponding to an overall survival of 106 months from the initial diagnosis and 74 months from biopsy-confirmed extrathoracic metastasis.

## 3 Discussion

Small-cell lung cancer (SCLC) is a highly aggressive malignancy characterized by rapid progression and a strong propensity for recurrence, and patients with relapsed disease continue to have poor clinical outcomes despite standard treatment. Here, we report a patient with initially limited-stage SCLC who underwent concurrent chemoradiotherapy followed by multiple subsequent lines of treatment, including irinotecan, pemetrexed, and anlotinib. The patient initially achieved disease control with anlotinib but subsequently developed right submandibular lymph node metastasis, indicating disease progression. Sintilimab, a programmed cell death protein 1 (PD-1) inhibitor, was therefore added to ongoing anlotinib treatment, and radiologic response was achieved after three cycles. Sintilimab was subsequently discontinued because of immune-related hypothyroidism, while anlotinib was continued as maintenance therapy. At the latest follow-up, the patient remained alive, with an overall survival (OS) of 106 months from the initial diagnosis. Such prolonged disease control is highly uncommon in patients with multiply relapsed SCLC and may suggest an unusual sensitivity to treatment and potentially distinct tumor biology.

### 3.1 Persistent antitumor activity of anlotinib may have provided an important foundation for long-term benefit

Anlotinib is a multitarget tyrosine kinase inhibitor that simultaneously inhibits several signaling pathways, including vascular endothelial growth factor receptors (VEGFRs), fibroblast growth factor receptors (FGFRs), platelet-derived growth factor receptors (PDGFRs), and c-Kit, thereby exerting antitumor effects through inhibition of tumor angiogenesis and tumor proliferative signaling. The ALTER1202 trial demonstrated that anlotinib improved clinical outcomes in patients with SCLC who had received multiple prior lines of treatment, with a median progression-free survival (PFS) of 4.1 months and a median OS of 7.3 months (6). In the present case, however, the duration of disease control achieved with anlotinib was markedly longer than that typically reported in previous studies.

Importantly, anlotinib in this case was not merely used as a companion drug during immunotherapy but had already demonstrated clear antitumor activity before sintilimab was introduced. The patient initially achieved disease control with anlotinib and subsequently developed right submandibular lymph node metastasis. Nevertheless, after sintilimab was added to ongoing anlotinib treatment, the patient again achieved a radiologic response and subsequently maintained anlotinib monotherapy after discontinuation of sintilimab. Thus, the clinical course suggests that the sustained antitumor activity of anlotinib may have provided an important foundation for the patient’s long-term disease control.

One possible explanation is that the tumor may have exhibited an unusual dependence on angiogenesis-related signaling pathways and therefore may have retained sensitivity to inhibition of VEGF/VEGFR and related pathways. By simultaneously targeting multiple pro-angiogenic pathways, anlotinib may have continuously restricted tumor vascular support and proliferative signaling, thereby delaying disease progression (5, 6). However, because molecular profiling was not available in this case, the biological basis underlying this prolonged sensitivity remains unclear.

### 3.2 PD-1 blockade may have reactivated antitumor immunity after disease progression

In addition to the sustained antitumor activity of anlotinib, the addition of sintilimab may have played an important role in reactivating antitumor immunity after disease progression. The PD-1/programmed death-ligand 1 (PD-L1) axis is a major mechanism of tumor immune evasion, and PD-1 blockade can relieve T-cell inhibition and restore the antitumor activity of CD8+ T cells.

In this case, the development of right submandibular lymph node metastasis during anlotinib treatment indicated breakthrough disease progression despite ongoing anti-angiogenic therapy. Sintilimab was therefore added to continued anlotinib treatment, after which the patient achieved a radiologic response following only three cycles of therapy. This clinical observation suggests that PD-1 blockade may have further enhanced antitumor immunity in the setting of ongoing anti-angiogenic treatment and may, at least in part, have overcome resistance to the preceding treatment strategy.

The patient also developed immune-related hypothyroidism during sintilimab treatment. Although immune-related adverse events should not be interpreted as direct evidence of antitumor efficacy, this finding indicates that clinically relevant immune activation occurred during PD-1 blockade. Theoretically, T-cell activation induced by PD-1 inhibition may persist beyond treatment discontinuation through the development of immunologic memory, potentially contributing to continued antitumor activity. However, evidence for durable benefit after discontinuation of immune checkpoint blockade has been derived mainly from immunologically responsive malignancies such as melanoma, whereas evidence in SCLC remains limited (8). Therefore, whether sintilimab independently contributed to the prolonged disease control observed in this patient remains uncertain.

### 3.3 Anlotinib and PD-1 blockade may exert complementary effects through vascular–immune remodeling

Beyond the independent antitumor effects of anlotinib and sintilimab, the clinical course of this patient raises the possibility of a complementary interaction between anti-angiogenic therapy and PD-1 blockade. Tumor angiogenesis and immune evasion are closely interconnected processes. Abnormal tumor vasculature can promote tissue hypoxia, restrict immune-cell infiltration, and contribute to an immunosuppressive tumor microenvironment through VEGF-related signaling (9).

Preclinical evidence suggests that anlotinib may facilitate tumor vessel normalization and remodel an immunosuppressive tumor microenvironment toward a more T-cell-inflamed phenotype. In a neuroblastoma model, anlotinib promoted vascular normalization and reprogrammed the immunosuppressive tumor microenvironment toward an immunostimulatory state, while combination with PD-1 blockade prolonged vascular normalization and enhanced tumor regression (9). These findings provide mechanistic support for the possibility that anlotinib may enhance the activity of PD-1 blockade by simultaneously modulating the tumor vasculature and immune microenvironment.

Conversely, PD-1 blockade may further reshape the tumor immune microenvironment. By relieving PD-1-mediated T-cell suppression, sintilimab may enhance the activity of CD8+ T cells and other effector immune populations. The resulting immune activation may, in turn, interact with the vascular microenvironment and potentially reinforce the effects of sustained anti-angiogenic therapy. Thus, the combination of anlotinib and sintilimab may not simply represent the additive effects of two agents but may instead involve coordinated modulation of both the tumor vasculature and immune microenvironment.

Clinical evidence also supports the potential activity of this combination in relapsed SCLC. In a phase II investigator-initiated study of sintilimab plus anlotinib as second- or further-line therapy for extensive-stage SCLC, the regimen achieved a median PFS of 6.1 months and a median OS of 12.7 months, indicating clinically relevant antitumor activity in previously treated patients (10). These findings provide clinical support for further investigation of the vascular–immune interaction between PD-1 blockade and anti-angiogenic therapy in SCLC.

The clinical course of our patient is consistent with this mechanistic hypothesis. The patient initially achieved disease control with anlotinib but subsequently developed right submandibular lymph node metastasis. After sintilimab was added to continued anlotinib treatment, the patient achieved a renewed radiologic response. Notably, sintilimab was administered for only three cycles and was subsequently discontinued because of immune-related hypothyroidism, whereas anlotinib was continued as maintenance therapy, with disease control subsequently maintained for a prolonged period. This course raises the possibility that short-course PD-1 blockade induced an immune response that interacted with sustained anti-angiogenic treatment and contributed to subsequent long-term disease control.

Accordingly, this case supports a potential therapeutic model in which anlotinib first inhibits abnormal angiogenesis and helps create a more permissive tumor microenvironment for immune activation; PD-1 blockade then enhances antitumor immunity and may further remodel the tumor microenvironment, thereby allowing continued anti-angiogenic therapy to exert more durable antitumor effects. This potential “vascular normalization–immune activation–sustained anti-angiogenic therapy” feedback loop may represent one possible explanation for the exceptionally prolonged disease control observed after multiple lines of treatment.

Nevertheless, these mechanistic interpretations remain hypothesis-generating. Because tumor tissue, immune-cell infiltration, and relevant molecular biomarkers were not dynamically assessed before and after treatment, we cannot directly demonstrate that such microenvironmental changes occurred in this patient or determine the respective contributions of anlotinib, sintilimab, and their potential interaction to the long-term outcome.

### 3.4 Limitations and future perspectives

This case has several limitations. First, as a single-case report, it cannot establish a direct causal relationship between the treatment strategy and prolonged survival, and the possibility that favorable intrinsic tumor biology contributed to the outcome cannot be excluded. Second, anlotinib was continued before and after the introduction of sintilimab, making it difficult to disentangle the relative contributions of sustained anti-angiogenic activity, PD-1 blockade, and their potential interaction. In particular, the patient had already demonstrated sensitivity to anlotinib before sintilimab was introduced; therefore, the subsequent long-term survival cannot be attributed solely to the addition of immunotherapy.

In addition, the patient was diagnosed in 2017, when molecular classification and biomarker testing in SCLC had not yet become routine clinical practice. Consequently, this case lacks information on PD-L1 expression, tumor mutational burden (TMB), molecular subtypes of SCLC, including ASCL1, NEUROD1, POU2F3, and YAP1, DLL3 expression, and characteristics of the tumor immune microenvironment (11, 12). The absence of these data limits our ability to further investigate the biological basis of the patient’s exceptional treatment sensitivity. Future studies incorporating comprehensive molecular profiling, immune microenvironment characterization, and dynamic biomarker monitoring may help identify patients who are most likely to derive durable benefit from combined anti-angiogenic and immune checkpoint blockade.

### 3.5 Conclusion

In conclusion, we report a patient with multiply relapsed SCLC who developed disease progression during anlotinib treatment, achieved a renewed response after the addition of sintilimab, and subsequently maintained long-term disease control with anlotinib maintenance after discontinuation of sintilimab, resulting in an OS of 106 months from the initial diagnosis. This case suggests that sustained anti-angiogenic activity of anlotinib, PD-1 blockade-induced immune activation, and a potential vascular–immune interaction between the two treatments may have jointly contributed to the prolonged disease control. However, given the inherent limitations of a single case, the relative contribution of these mechanisms cannot be determined. Prospective studies are warranted to define the optimal patient population, treatment sequence, duration of immunotherapy, and predictive biomarkers for anti-angiogenic therapy combined with immune checkpoint blockade in relapsed SCLC.

## Patient perspective

The patient reported that she was able to maintain most daily activities throughout long-term treatment and follow-up. She considered the overall treatment burden acceptable. After levothyroxine replacement, hypothyroidism did not substantially interfere with her daily life. She regarded the prolonged disease control achieved after sintilimab plus anlotinib and subsequent anlotinib maintenance as clinically meaningful.

## Data Availability

The original contributions presented in this study are included in the article. Further inquiries can be directed to the corresponding author.
